# RealTalk evidence-based communication training resources: development of conversation analysis-based materials to support training in end-of-life-related health and social care conversations

**DOI:** 10.1186/s12909-022-03641-y

**Published:** 2022-08-23

**Authors:** Ruth Parry, Becky Whittaker, Marco Pino, Laura Jenkins, Esme Worthington, Christina Faull

**Affiliations:** 1 Centre for Research in Communication and Culture, School of Social Sciences and Humanities, Loughborough University, Loughborough, LE11 3TU UK; 2 LOROS Hospice Leicester, University Hospitals of Leicester and Honorary Professor University of Leicester, Leicester, LE3 9QE UK

**Keywords:** Communication training, Intervention development, Teaching, Education, Health occupations, Video recording, Conversation analysis, Palliative care, End-of-life care

## Abstract

Training to enhance healthcare practitioners’ capabilities in engaging people in sensitive and end-of life-related conversations is in demand. However, evaluations have either not measured, or found very limited impact on actual practice and patient experience. Training effectiveness is improved when it is based on in-depth evidence, reflects the complexity of real-life interactions, and instils principles adaptable to everyday practice. A relatively new source of in-depth evidence and practice-relevant insights on healthcare interactions is conversation analytic research, a form of observational analysis of real-life interactions. However, conversation analytic research findings have largely been disseminated by and for scientists, rather than clinicians and trainers. We used conversation analytic evidence to develop resources for use by healthcare trainers. The aim was to increase training’s evidence-base and authenticity. We further aimed to develop resources applicable to working with learners ranging from novices to advanced practitioners.

**Methods: **Using an intervention development approach, we created online video-clips and supplementary written materials for professionals who deliver training, supervision, and support in healthcare communication for staff and students. The materials were reviewed by an advisory group comprising clinicians, lay consultees, educators, and researchers, and piloted by trainers in UK universities, NHS organisations and independent hospices. We refined materials based on their feedback.

**Results:** The resulting ‘RealTalk’ resources focus on practices for communicating with patients and their companions about end-of-life and prognosis. Two core training modules were developed, each comprising several patient case studies featuring video-clips from real-life healthcare consultations. The clips featured practices that patients and experienced practitioners use in approaching end-of-life matters. The case studies also included evidence-based descriptions of observable practices and the principles underlying these, alongside transcripts and case synopses.

**Conclusions:** RealTalk training resources aim to facilitate evidence-based, experiential and reflective learning, focusing on communication challenges, practices and principles for end-of-life-related interactions. The resources are designed for use by trainers for delivering all levels of training, from introductory to advanced, in both formal and informal training settings. Our development process may serve as a blueprint for the production of future evidence-based training resources based on conversation analytic research.

**Supplementary Information:**

The online version contains supplementary material available at 10.1186/s12909-022-03641-y.

## Background

We report our development of a set of evidence-based online resources, we term these ‘RealTalk’. They comprise video-clips from unobtrusively recorded, naturally-occurring healthcare consultations and accompanying written materials. We aimed to develop resources that could be used within training (both formal and informal) by health and social care professionals charged with delivering training, supervision, and support to healthcare practitioners and students. To date, RealTalk’s focus has been on sensitive, important conversations related to end-of-life. In the first version, reported here, we produced resources centred on two aspects of these conversations: facilitating talk about end-of-life and related concerns, and discussing prognosis.

The resources are based upon evidence and insights from published conversation analytic research. Conversation analysis is a rigorous and radically empirical approach to studying language and social interaction, its data comprise (recorded) naturally-occurring interactions. Its findings comprise detailed insights into the challenges we face in our interactions with one another, and detailed description of the structures and the functioning of the numerous practices – verbal and bodily – that we use in order to navigate these challenges and ‘get things done together’ through our interactions. Consistent with conversation analytic evidence and insights, RealTalk’s focus is on principles and practices, which learners can then apply and adapt in individual circumstances. That is, RealTalk does not teach ‘atomised’ communication skills and scripts.

We intended that RealTalk would provide trainers with an accessible means to update and increase the evidence-base and the authenticity of their training. Given that patients who are likely to be in their last year of life attend a wide variety of healthcare settings, we aimed to make resources applicable to a wide range of learners – from highly experienced specialists to students and novices.

Besides reporting specifically on RealTalk, we hope our paper will also provide a blueprint for future development of communication training resources based upon conversation analytic data, findings, and insights.

We begin by explaining the rationale for developing new resources in what is already a crowded scene, then we describe methods we used to develop and pilot RealTalk. Next we describe the structure and content of the resulting RealTalk resources, followed by discussion of our findings and the limitations of our approach.

### Communication training: room for improvement

Both w*hat* healthcare practitioners communicate and *how* they do so can significantly influence patients’ behaviours, satisfaction, attendance, clinical outcomes, and rates of complaints [1]. Healthcare practitioners’ communication practices are not innate and fixed characteristics: there is evidence that some specific practices for particular contexts can be acquired via appropriate training [2–4]. Training in healthcare communication is thus in demand, particularly in relation to patient-centred communication (see [5] which outlines different levels or tiers of conversation capabilities required according to practitioners’ roles), and in relation to engaging patients and their companions in important, sensitive conversations relating to the end-of-life.

Studies evaluating the effects of training are limited in both quality and quantity. Systematic reviews of randomised controlled trails of communication training for practitioners in cancer care [6–8] and other areas of healthcare [9–11] have found, at best, only small effect sizes on practitioner communication and patient outcomes and only limited transfer into practice. Importantly, such trials and reviews of communication training assume that quantification of individual communication skills is a valid means of measuring impacts of training, However, arguments developed through critically reviewing current research, [9] and practice [12], make a convincing case that it is often impossible to meaningfully atomise complex behaviours, such as conducting consultations or building relationships, into component skills [12]. This recognition is consistent with conversation analytic evidence that our communication with one another works through sequences - “what one participant says and does is generated by, and dependent upon, what the other has said and done” ([13]. p59) This sequential structure is a key resource by which people understand one another - because each spoken turn inherently shows how that speaker has interpreted what has been said and done so far [14]. Attempting to measure changes in frequency of decontextualised ‘skills’ displayed by an individual speaker is thus rarely a valid way of measuring change, and thus that different approaches, including observational and qualitative ones must be used. Another likely contributor to findings of limited effectiveness is that existing training largely relies on weak indirect evidence – what people can report about communication, rather than direct observation of real-life interactions.

### Fields of knowledge relevant to improving healthcare communication training

Evidence and insights relevant to improving training can be found in critical reviews of healthcare training [11, 15] including those which draw or focus on academic research in education [9] and in clinical and training psychology [10, 12, 16]. These have found that the effectiveness of training, including its transfer into practice is enhanced when training has content that reflects the complex, context-specific nature of actual interactions [11, 12], and is closely relevant to trainees’ real-life practice [15]. Also, where training is focused upon complex skills (such as building empathy, or raising and discussing delicate topics), the objective should be “to get the learner to inculcate generalizable rules, concepts, and principles [so that they can] formulate their own plan for how to apply those rules and customize” what they have learned to fit their own needs and circumstances ([16] p1069), rather than teach trainees to basically mimic a procedure that is trained (a more suitable approach in, for instance, training in a technique for cleaning a particular instrument).

We also know that to be effective, communication training needs to include a range of experiential and interactive components [10, 15]. In current healthcare communication training, where such experiential and interactive components are used, these often involve role-play scenarios and simulations. However, recent empirical comparative research has shown that role-play and simulation differ in systematic and important ways from authentic, real-life interaction and its demands [17–19]. Clearly this is problematic, given – as noted above - that for communication training to be effective and transferable, it needs to closely reflect real-life practice and the complexity of actual interactions. Furthermore, role-play has been criticised for failing to help learners develop the analytical capacity and flexible strategies crucial to real-life, high-stakes situations [20]. Role-play and simulation in communication training can also be time-consuming, expensive, stressful for learners, and place high demands on trainers. A final component known to be important in enhancing training is its founding in robust, in-depth evidence, [9, 10] and we will now move to a key source of such evidence.

Conversation analytic research is uniquely placed to provide robust, in-depth evidence. This field has grown rapidly since the mid-1970s [21]. It has generated a substantial body of findings relevant to the content and effectiveness of healthcare communication training. Nonetheless, this field is not yet well known amongst trainers, practitioners, and policymakers [22].

Conversation analytic studies provide knowledge about interactional practices entailing language, other vocal sounds, and other bodily behaviours, understandings derived from systematic analysis of recorded real-life interactions. By collecting and analysing multiple episodes of specific communication tasks and sequences, conversation analysts produce detailed evidence on the structure, functioning, and consequences of communicative behaviours. Importantly for training, conversation analytic findings include detailed descriptions of the structure and function of communication practices that are normally tacit – things we do but are not conscious of, and thus could not describe.

Several published studies have reported using conversation analytic findings as the basis for interventions [23]. Results from evaluations of conversation analysis-based training interventions have been very promising, finding statistically significant changes in practitioners’ communication during actual practice [2, 3, 24–28]. To date, however, interventions based on conversation analysis have largely targeted highly specific communication practices, which trainees have been taught to closely replicate (e.g., specific question wording for specific moments within consultations), [3, 27] and some have required that training interventions be delivered by experienced (scarce, and expensive) conversation analysts [26, 28].

Given these insights and findings, we developed new conversation analysis-based training resources: ‘RealTalk’, which are designed to be used by professionals who deliver training in communication relevant to end-of-life and palliative care. Our aim was to develop resources that would:

- Increase the authenticity, relevance, and empirical evidence-base of communication training for health and social care staff and students.
- Facilitate learning and reflection on complex, multifaceted communication tasks, whilst avoiding the shortcomings of role-play and simulation.
- Be accessible to trainers and trainees without requiring them to have expertise in conversation analysis, and thus relatively easy and economical to disseminate to trainers in those public sector and charitable organisations which offer communication training.
- Be suitable for trainers to use ‘off the shelf’, with written materials providing extensive guidance on using the resources.
- Be usable and relevant for a wide range of formal and informal training facilitated by diverse trainers, in diverse settings, and for diverse participants.

## Methods

### Identifying aspects of end-of-life related conversations upon which to focus

At the beginning of our development process, we decided to focus on interactional challenges, practices and principles associated with promoting end-of-life talk, and on conversations about prognosis. We made this decision on the basis of the analyses emerging from our underpinning research (described below), and also:

- Reviews of qualitative studies about the preferences and perspectives of patients, companions and practitioners [29, 30];
- Priorities articulated by the UK’s National Palliative and End-of-life Care Partnership [31];
- Discussions with educator and clinical colleagues at four large UK hospices, all of whom had extensive experience of running communication training programmes and of supporting practice-development;
- Discussions within the research team and with our project advisory group (which comprised lay consultees, academics, educators, and clinicians).

### Conducting underpinning analyses

We developed the training resources as part of our ‘VERDIS’ research programme. VERDIS comprises a series of interlinked conversation analytic studies of communication in supportive and palliative care [32]. These studies included examining ways in which doctors facilitate talk about end-of-life and how patients respond, [33] and how patients ask, and doctors respond to, questions about remaining life expectancy [34]. Our research involved collecting and analysing video-recordings of inpatient and outpatient consultations that took place within a large UK hospice. The dataset comprised recordings of 37 consultations, involving five experienced palliative medicine doctors, 37 terminally-ill patients, and 17 accompanying companions. All participants had provided written informed consent.

We used conversation analysis, described above, to study the video data. We searched the full dataset for relevant episodes, then worked with detailed transcripts and the recordings themselves in order to closely examine patterns in relation to when such episodes arise, how the sequences within them are structured and how they function. As is required in conversation analysis, [22] our own analyses drew and built upon previously established conversation analytic findings.

The recordings we had made were of naturally-occurring consultations rather than simulations of any perceived ‘ideal’ practice. However, the consultations involved highly trained, experienced palliative medicine specialists. They thus featured numerous examples of practices that help to sensitively and effectively navigate key challenges in end-of-life-related conversations.

### Compiling the resources

**Fig. 1:**
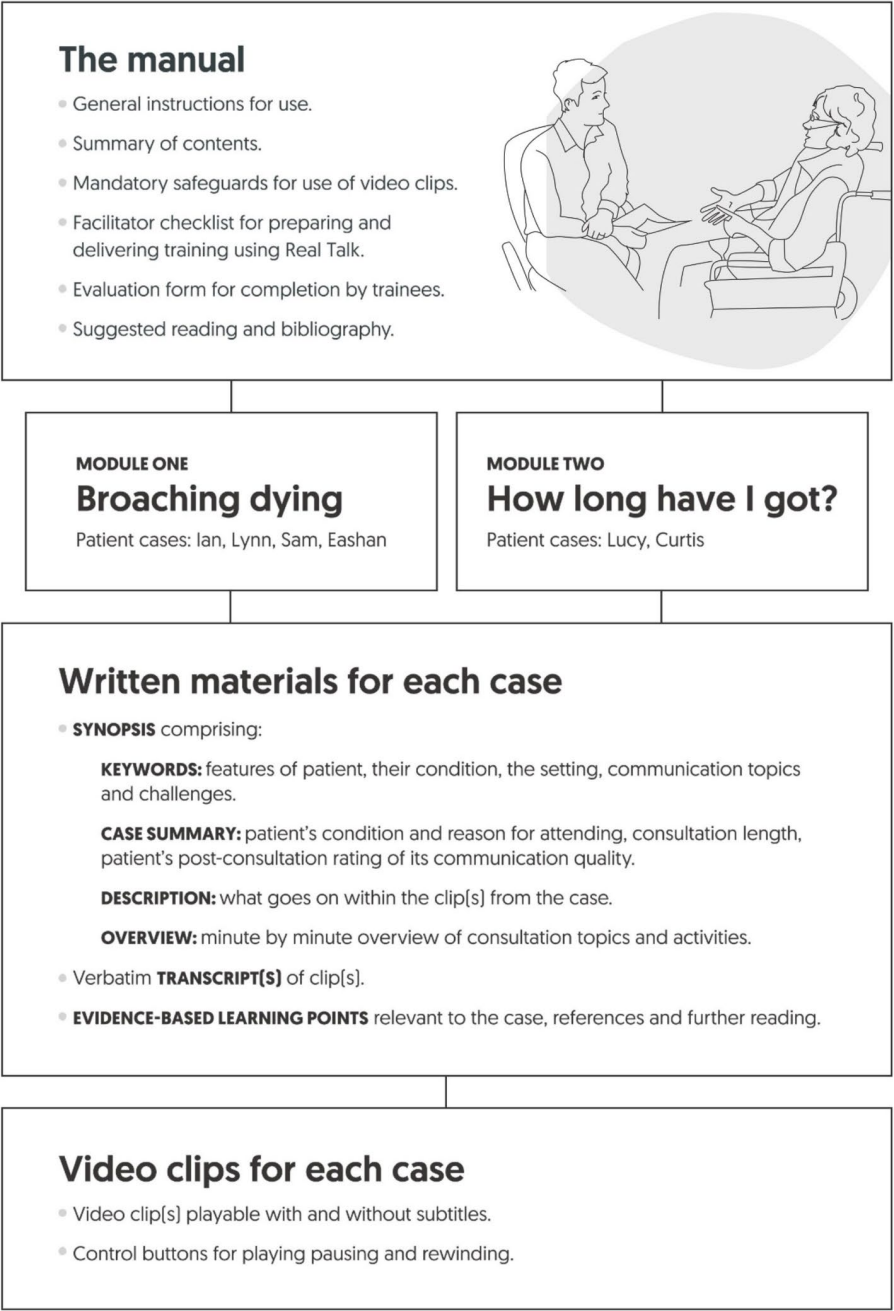
Overview of the RealTalk training resources’ structure and contents

Having decided to focus the RealTalk resources on prognosis conversations and on practices that facilitate talk relating to end-of-life, we selected a series of recorded consultations from the VERDIS dataset that included relevant episodes which clearly illustrated key practices. We created a case study from each selected consultation (an outline of the case studys' content is included in Fig. 1). One or more video-clips formed the core of each case. We compiled three written documents for each case. First, a series of learning points which described and explained the communication practices, challenges and principles evident in the video-clip(s). Learning points were based on our own analyses, [33, 34] and other conversation analytic evidence, including [35–40]. Second, a case synopsis provided context for the clips by including details about the patient, and a summary of what happened over the course of the full consultation. Third, we created verbatim transcripts of the video-clip(s) featured in the case. We shared drafts with an advisory group of clinicians, lay consultees, educators, and researchers, sought feedback, and modified accordingly.

Working with technical teams specialising in digital educational materials, [41] branding and web-design, [42, 43] and our project advisory group who gave feedback and advised on modifications, the resulting written documentation was compiled within a comprehensive written manual (Fig. 1 outlines the Manual’s content) [43], and all materials, including the video-clips, were housed on a password-protected website (www.realtalktraining.co.uk), designed to be accessed only by eligible registered parties (Eligibility criteria are provided in Additional File Part One).

In the clips, all verbal references to people or places were edited out but people’s faces and bodies were still visible. Safeguards for the video-clips and their use were drawn up, based on a literature review [44] and an empirical interview study, [45] These safeguards are provided in Additional File, Part Two. Trainers were required to agree to uphold and to share the safeguards with trainees before showing any video-clips.

### Piloting RealTalk resources

Experienced clinical trainers were identified and recruited from NHS organisations, universities and hospices, via meetings, conferences, and organisational newsletters, and through direct contact with individuals known to the project team and advisory group.

The trainers were asked to first familiarise themselves with the materials, and then use them to prepare and deliver at least one formal training event. Several of these training events were observed directly (by author BW) as part of a qualitative evaluation of the acceptability and utility of the RealTalk resources (reported elsewhere [32, 46]), and feedback was collected from trainers and trainees following the training events. The observations and feedback were used to further optimise the resources before launching RealTalk.

## Results

An overview of the structure and contents of the finalised RealTalk resources is provided in Fig. 1. We produced two core modules: ‘Broaching dying’ and ‘Life expectancy enquiries’. Each case in the modules was named with a pseudonym of the patient’s name. The finalised resources were organised, in response to the feedback we gathered, such that individual components, cases, or communication-related themes and materials could be selected by trainers, thereby supporting them to identify and fit the resources to specific training episodes. Keywords for each case were important in enabling trainers to select relevant cases and materials.

As noted above, the finalised product also included a Manual [42] comprising not only the written documents for each case study, but also sections on ‘How to use RealTalk’, we provide key parts of this guidance in Additional File Part Three). We printed and bound the Manual and sent it to eligible trainers who registered to use RealTalk and agreed to the safeguards (Additional File Part Two).

Our own use of RealTalk, feedback from our pilot trainers, and informal feedback from trainers who registered for the finalised resources all indicate that RealTalk authentically illustrates and provides novel learning points on a diverse range of communication challenges and practices. Table 1 provides examples.

**Table 1:** Examples of some of the communication practices featured in the RealTalk training resources

• Ways to encourage patients to talk (more), including use of body posture, gaze, and silence in ways that convey that the practitioner is ceding the ‘conversational floor’ to the patient. • Empathic behaviours, including tone of voice, gestures and facial expressions, explicitly naming emotions, and practices that convey that one has insight and understanding whilst avoiding implying complete understanding of the patient’s own unique experience. • Active listening – that is, encouraging the patient to say more and conveying that one is carefully attending, practices include use of body movement, gaze, and continuers (e.g. Mm, and Uhuh) in ways that show that the ‘conversational floor’ is the patient’s. • Summarising both in the midst of, and towards the end of, consultations; how summaries can foreground particular matters, and can also be used to encourage talk about as yet undiscussed matters. • Facilitating deepening disclosure in a careful and sensitive manner, including through follow-up questions and other cautious step-by-step moves towards more explicitly mentioning end-of-life concerns. • Providing patients with opportunities to raise questions and concerns, in ways that encourage them to do so, and that steer them towards raising particularly sensitive matters about the future and end-of-life. • Seeking patients’ perspectives and understandings before providing prognostic or other potentially distressing information, and providing that information in ways that take into account the patient’s perspective.	

Since launching the finalised resources via the RealTalk website, we have registered a wide range of trainers from diverse organisations, including universities, NHS Trusts and independent hospices, throughout the UK. The resources have been used in training designed for participants across a wide spectrum from specialist palliative care professionals through to students early in their studies of medicine and nursing. To date, more than 400 trainers have registered.

## Discussion

The value of the RealTalk training resources lies in their foundation of robust, in-depth evidence derived from close observation of naturally-occurring interactions. This conversation analytic evidence provides a level of knowledge and understandings that is not possible through indirect research – that is, studies which ask people about their communication practices and preferences. The resources are authentic and relevant to real-life practice, reflect the complex, context-specific nature of actual healthcare interactions, and integrate a range of experiential and interactive components.

Rather than recommending a specific model or script, or teaching a specific ‘skill’ to be replicated in practice, RealTalk facilitates reflective learning of principles and practices which can guide and be adapted to the individual and complex circumstances and conversations which practitioners face in healthcare conversations (an approach consistent with ‘open skills training’ [16]). In line with the conversation analytic approach to understanding and analysing interaction, trainers using RealTalk are encouraged to ask trainees to avoid making evaluative judgements about the conversations within the video-clips, and instead to describe what they observe by asking: “What did you see?” and “What did you hear?” (related guidance for trainers on working with RealTalk resources can be found in Additional File Part Three).

Despite being grounded in findings derived from close and rigorous analyses of interaction by experienced conversation analysts, the RealTalk resources do not require that trainers themselves have expertise in the concepts, tools, and previously established findings of conversation analysis. Our aim was for the resources to be suitable for use ‘off the shelf’, without necessitating prior specialist training. Since launching the RealTalk resources, feedback has indicated that trainers find training workshops focused on how to use the resources useful, but that prior training is not essential for RealTalk users. A series of ‘train the trainer’ events we have run since our launch has been positively received [46].

Feedback indicates the resources are flexible enough to be used in delivering both formal and informal communication training for health and social care professionals and students, and are applicable to a wide range of learners – not just highly experienced specialists, but also students and novices.

Since the resources were first developed, we have created additional content relating to end-of-life communication, including communicating about pain symptoms, conveying empathy, and bereavement support interactions. In response to the coronavirus pandemic, we have also added processes and guidance on using RealTalk in remote online training.

Our development process and the resulting resources have limitations. The resource development spanned 3 years, and was therefore costly, but feasible thanks to funding from both the host universities and an external Health Foundation grant. The project advisory group and piloting trainers were not paid for familiarising themselves with the draft materials or providing feedback, though some patient and public involvement advisory group members were paid for their time. Further costs included fees for web design and hosting, and printing and postage of the Manual. We have since slightly reduced the running costs by replacing this printed resource with online files which trainers can opt to print for themselves. Another limitation is that conditions of our ethical approval stipulated that the video-clips wherein patients' and their companions' faces are visible may not be used in training outside the UK; this limits the pool of potential users. However, an international version, in which faces in the video-clips are disguised, will be launched shortly. Another potential limitation is that we cannot know whether and how much adaptation of our process and the resources’ structure would be needed conversation analysis-based resources for contexts outside end-of-life related conversations. Finally, the effectiveness of RealTalk in enhancing trainers’ or trainees’ practice should not be assumed, it would need to be evaluated (with the inherent challenges of doing so [9, 12]).

We hope that, the process by which we developed RealTalk and the resulting training resources themselves, will provide a model for the development of other communication training resources using data and findings from conversation analytic research.

## Conclusions

The RealTalk resources encompass learning points from cutting-edge conversation analytic communication research and video-clips showing real life healthcare conversations in all their complexity and authenticity. Our development of RealTalk provides a blueprint that may be used by others in developing training resources that draw on conversation analytic evidence generated from recorded real-life interactions.

## Supplementary Information

**Additional file 1.**

## Data Availability

The video and audio recorded datasets analysed during the current study are personal data, entailing healthcare episodes, and are not publicly available under the conditions required and agreed by the UK National Research Ethics Service Committee (now renamed the Health Research Authority). Data are however available from the authors upon reasonable request provided the requesting person(s) have formal Health Research Authority (or equivalent ethical approvals organisation outside England) permission to use the data. The RealTalk video-clips and accompanying written materials, generated during the current study, are also not publicly available due to conditions required and agreed by the UK National Research Ethics Service Committee (now renamed as the UK Health Research Authority). These data are however available to communication skills trainers who are eligible for and registered via the RealTalk communication training resources platform. Trainers can apply for access via https://www.realtalktraining.co.uk/apply.

## Abbreviation

VERDIS: Video-based communication research and training in interactions entailing decision-making, empathy, and pain in supportive and palliative care
