# Supplementary material for: RealTalk evidence-based communication training resources: development of conversation analysis-based materials to support training in end-of-life-related health and social care conversations
Source: BMC Med Educ. 2022 Aug 23;22:637. doi: 10.1186/s12909-022-03641-y (PMC9395846; doi:10.1186/s12909-022-03641-y)
Supplement: Supplementary file 1 — Additional file 1. [file 12909_2022_3641_MOESM1_ESM.docx]

**ADDITIONAL FILE**

**Part One: Eligibility for registration to use the RealTalk video-clips and resources in formal and informal training**

The research protocol and the participant consent process stated that eligible trainers should:

1. Use the resources for training within the UK
2. Provide training for staff and students in health and/or social care
3. Provide training in communication practice, including end-of-life care-related communication
4. Not make a profit from their training work

**Part Two: Safeguards**

TRAINERS MUST AGREE TO UPHOLD THE FOLLOWING WITH REGARDS THE VIDEO-CLIPS

The video-clips are only to be used for the purposes of preparing and delivering face-to-face training for professionals working with patients, and students planning to work with patients who are accessing NHS and hospice services

1. Video-clips are not to be shown outside of the UK, Ireland and the Channel Islands
2. You must not financially gain as an individual from your access to the video-clips
3. Video data are not to be reproduced in whole or in part nor should you attempt to download them from the website
4. You must agree to include the RealTalk Safeguards for Trainees in every teaching session delivered
5. Video-clips are only to be used by the named trainer, who must agree to keep the RealTalk project team informed of any move to a different organisation

Copyright © 2019 Loughborough University. All rights reserved.

TRAINEES MUST AGREE TO UPHOLD THE FOLLOWING WITH REGARDS THE VIDEO-CLIPS

1. These materials include content that can be distressing; feel free to step out of the session if you need to
2. It is possible that in your working or personal life you could come across the people in the videos. Be alert to the fact that these people are unlikely to know who has seen the videos
3. At no point during or after the training should you talk about any individual in personal or negative terms
4. You must not take any photographic images nor make audio or film recordings of the clips or any part thereof
5. No one in the recordings claims that their practice is perfect, however the clips include skills and actions that contribute to good practice
6. All the people involved gave their permission for clips from their recordings to be used in training on the proviso that these safeguards are upheld

Copyright © 2021 Loughborough University. All rights reserved.

*Since the work reported in this study, and given the effects of the pandemic, we have drawn up amended safeguards fitted to live, remote, online training.*

**Part 3: Sections from the Manual’s ‘How to use’ guidance for RealTalk trainers**

OVERALL PERSPECTIVE ON COMMUNICATION

Any task that gets done through communication, be it asking for information, or encouraging someone to talk about their own dying and death, can be done in a variety of ways. Each of the different ways will have its pros and cons. This understanding recognises that there is never (or rarely) only one optimal way of doing a healthcare communication task or dealing with a communication problem. Instead, for any particular set of circumstances, some ways will be more appropriate and effective than others.

RealTalk is designed to help trainees examine various communication tasks and problems, help them consider different ways of handling these, and the various pros and cons of different ways of handling them.

The conversation analytic perspective focuses on what people do, attempt, and accomplish through their communicative actions. It focuses on what is observable and evident in how people communicate, not on under-the-surface matters like intentions and motivations (this does not mean we don’t have intentions and motivations, it is just that conversation analysis does not give us access to these). An important assumption in conversation analysis is that people do what they do for good reasons, which may not always be obvious without close analysis. This perspective provides a constructive way of working with recordings.

WHAT REALTALK CONTAINS

At its core are video-clips from consultations recorded in the outpatient, inpatient and day therapy services of a UK Hospice and is divided into modules of learning.

Module One comprises Cases 1 – 4, which focus on ‘broaching dying’, i.e., how practitioners encourage talk about patients’ end-of-life. Broaching dying is a necessary step towards making plans for the future and provides opportunities to address feelings and fears.

Module Two comprises Cases 5 and 6, which focus on when patients ask, and doctors respond to, a ‘How long have I got?’ type of enquiry.

Trainers can use the clips to focus discussions on other topics, including empathy, active listening, body language, expressing compassion, or dealing with distress and uncertainty.

A day or more after each consultation, the patient was asked to rate communication in their consultation using a Care and Relational Empathy (CARE) questionnaire. The patient’s rating is given in the synopsis page for each case; it can be used to incorporate the patient’s perspective within the training. A copy of the CARE questionnaire can be viewed/downloaded here: <https://caremeasure.stir.ac.uk/> *[Accessed 5/5/22]*

MODULE ONE: ‘BROACHING DYING’ CASES

Each case comprises multiple clips detailing progressive movement from initial hints or opportunities to more direct talk about dying and about plans for the future.

*Case One - Ian*

Four clips.

Keywords: broaching dying, euthanasia, pain, mood, resuscitation, returning to something the patient has mentioned earlier, unpacking ambiguity, motor neurone disease, day therapy, advance care planning.

*Case Two - Lynn*

Four clips.

Keywords: broaching dying, breathlessness, low mood, fear, reluctance to talk about dying, returning to something the patient has said earlier, advance care planning, heart failure, long term cancer survivor, hospice outpatient, empathy.

*Case Three - Sam*

Five clips.

Keywords: broaching illness progression and dying, communication with relative present in the consultation, ventilator, reluctance to talk about dying, neurological condition, hospice outpatient.

*Case Four - Eashan*

Three clips.

Keywords: broaching dying, patient expectations, fear, communication with relative present in the consultation, returning to something the patient has said earlier, preferred place of death, patient reluctance to talk about dying, advance care planning, advanced cancer, hospice outpatient, empathy.

MODULE TWO: ‘HOW LONG HAVE I GOT?’ CASES

*Case Five - Lucy*

One clip.

Keywords: difficult questions, life expectancy, imminent dying, uncertainty, empathy, pain, distress, desire to die, advanced cancer, hospice inpatient.

*Case Six - Curtis*

One clip.

Keywords: difficult questions, life expectancy, uncertainty, advanced cancer, hospice inpatient

WAYS OF WORKING WITH THE VIDEO-CLIPS

RealTalk is designed to fit into and complement your existing ways of training. You will need to spend some time familiarising yourself with the materials and planning how to use them. We have provided broad suggestions for using the clips, rather than detailed lesson plans, because ways the clips can be used are very diverse and flexible. We have provided examples of the ways clips can be used. The following are some suggestions that may be useful in planning training using the resources.

*Introducing the clip(s)*

Before playing any video-clips remember that you must cover the Safeguards. We encourage you to provide the trainees with the context of the clip by drawing on the information we have provided in the case synopses.

Because so much goes on even in short episodes of communication, it is useful to ‘prime’ trainees in advance about particular practices or skills they are going to see or that you specifically want them to look out for.

Tell trainees that on watching a clip they are likely to feel some practices are good, and some less good, and that you are going to work on unpicking precisely what is going on that leads to such impressions.

In using a conversation analytic perspective, we encourage trainees to focus on what they see and hear, not – in the initial discussion of clips at least – on patients’ and doctors’ intentions, motivations and feelings.

*Viewing and discussing clips*

Video-clips can be played with or without subtitles. This will depend on how you are asking trainees to work with the clips.

When working with the recordings, you will find that it is really easy for a group to move rapidly into hypotheticals - how things could have been done differently, or into judgements - particularly critical ones about what the practitioner did or didn’t do.

We encourage you to avoid this. Instead, to maximise learning from the clips, we suggest you facilitate a “What I saw, what I heard” discussion before moving into discussion of what could be done differently. The suggested teaching and learning points we provide for each case are designed to support “What did you see?” and “What did you hear?” discussions. This encourages analytic, descriptive and comparative thinking*.*

We request that you discourage trainees from making moral evaluations of the practitioners, patients, and their communicative actions – in part because this is unlikely to be pedagogically useful, and in part because any such evaluations would be based on very short clips that can provide an incomplete picture of the whole consultation and practitioner/patient relationship.

*Starting and stopping the clip*

A section of the clip can be played, then stopped to promote discussion, before you play the next part. This is particularly helpful with the longer clips. A ‘stop and discuss’ approach can help facilitate discussion and/or a focus on a specific learning point.

*Modified role play*

This technique involves stopping a clip at a crucial moment (such as just after a patient has mentioned something salient or asked a particular question) and asking one or more members of the group to role play what they would do next. This approach can be less nerve-racking than conventional role play. It also allows you to follow up with examination of what actually happened in the real world.

This technique is a useful one for demonstrating that there are multiple ways of working with difficult conversation topics. It can also help allay concerns that some practitioners have about ‘doing it right’ or fear of ‘getting something wrong’.

*Transcripts*

We have provided transcripts for each clip, these can be printed out for use in training or shown on a screen. For example, a transcript can be provided before showing a clip to create discussion in pairs: what do trainees notice about language, turn taking, silences etc.? This can help to prompt thought and engagement prior to showing a clip. Transcripts are likely to be useful with more advanced learners, or in longer training where learners may be asked to write reflective pieces using the transcripts

*Facilitator notes*

We provide below a ‘facilitator crib sheet’ for use with any of the video-clips. Our experience is that it is useful for trainers to have pre-prepared notes on potential discussion topics, particularly if a group are slow in getting conversations started in class. The notes can be adapted to include your event learning outcomes.

REALTALK FACILITATOR CRIB SHEET

Remember, there is no single right way to communicate in difficult situations, these resources may help us when considering the different ways of doing so and their pros and cons. The conversation analytic perspective focuses on what people do, attempt, and accomplish through their communicative actions. People do what they do for good reasons, though this is not always obvious without close analysis. This is the focus for how you will use the video-clips with your trainees. To get the most out of using the video-clips we suggest the following:

*Plan the session*

1. Familiarise yourself with the learning points for the case you are using
2. You may wish to print the transcript for use in group work, and/or to include the transcript in your on-screen presentation materials

*Ensure the safeguards are explained before showing any video-clips*

1. Embed the slide we provide into your presentation
2. Discuss the safeguards with trainees before you show any of the video-clips

*Provide background to the case you are using*

1. Introduce each clip, provide the trainees with the context of the clip by drawing on the information in the case synopses
2. Prime trainees in advance about particular practices or skills they are going to see and hear. The clips are rich in multiple details, give them a steer about what they should focus on
3. Before showing a video-clip, tell trainees that after showing the clip you will be asking them “What did you see?” and “What did you hear?”

*Playing the video-clips*

1. You can play them with or without subtitles
2. Focus on what did happen, rather than what did not happen
3. Keep the focus of discussions on the strategies and practices you can see and hear
4. Keep it analytical - avoid negative critique of individuals
5. Check that the focus of discussions is on the process of communication, on how people navigate ways through sensitive conversations. Remember, the conversation analytic perspective focuses on what people do, attempt, and accomplish through their communicative actions

*Check trainee wellbeing*

Some trainees may find a clip distressing, particularly given their authentic nature.
